# Label-free imaging of human brain tissue at subcellular resolution for potential rapid intra-operative assessment of glioma surgery

**DOI:** 10.7150/thno.59244

**Published:** 2021-05-24

**Authors:** Defu Chen, David W. Nauen, Hyeon-Cheol Park, Dawei Li, Wu Yuan, Ang Li, Honghua Guan, Carmen Kut, Kaisorn L. Chaichana, Chetan Bettegowda, Alfredo Quiñones-Hinojosa, Xingde Li

**Affiliations:** 1 Department of Biomedical Engineering, Johns Hopkins University School of Medicine, Baltimore, Maryland 21205, USA.; 2 Institute of Engineering Medicine, Beijing Institute of Technology, Beijing 100081, China.; 3 Department of Pathology, Johns Hopkins University School of Medicine, Baltimore, Maryland 21205, USA.; 4 Department of Neurologic Surgery, Mayo Clinic, Jacksonville, FL 32224, USA.; 5 Department of Neurosurgery, Johns Hopkins University School of Medicine, Baltimore, Maryland 21205, USA.

**Keywords:** multiphoton microscopy (MPM), two-photon excited fluorescence (TPF), glioma, multiphoton endomicroscope, histopathology

## Abstract

**Background:** Frozen section and smear preparation are the current standard for intraoperative histopathology during cancer surgery. However, these methods are time-consuming and subject to limited sampling. Multiphoton microscopy (MPM) is a high-resolution non-destructive imaging technique capable of optical sectioning in real time with subcellular resolution. In this report, we systematically investigated the feasibility and translation potential of MPM for rapid histopathological assessment of label- and processing-free surgical specimens.

**Methods:** We employed a customized MPM platform to capture architectural and cytological features of biological tissues based on two-photon excited NADH and FAD autofluorescence and second harmonic generation from collagen. Infiltrating glioma, an aggressive disease that requires subcellular resolution for definitive characterization during surgery, was chosen as an example for this validation study. MPM images were collected from resected brain specimens of 19 patients and correlated with histopathology. Deep learning was introduced to assist with image feature recognition.

**Results:** MPM robustly captures diagnostic features of glioma including increased cellularity, cellular and nuclear pleomorphism, microvascular proliferation, necrosis, and collagen deposition. Preliminary application of deep learning to MPM images achieves high accuracy in distinguishing gray from white matter and cancer from non-cancer. We also demonstrate the ability to obtain such images from intact brain tissue with a multiphoton endomicroscope for intraoperative application.

**Conclusion:** Multiphoton imaging correlates well with histopathology and is a promising tool for characterization of cancer and delineation of infiltration within seconds during brain surgery.

## Introduction

Surgical resection is first-line treatment for many cancers. Maximizing cancer resection while sparing adjacent healthy tissue is crucial for optimal treatment outcomes 1, 2. Consequently, intraoperative techniques for identification of cancerous versus non-cancerous tissues are essential.

Frozen sections and smears are the most commonly used techniques for intraoperative assessment during cancer surgery. These methods typically require 20-30 minutes to process, in contrast to the standard formalin-fixed, paraffin-embedded (FFPE) histopathology which usually requires >24 hours. However, the freezing process is destructive to tissue and may introduce image artifacts 3, and the smear preparation essentially eliminates information about cytoarchitecture. Both techniques are also subject to sampling errors, as it is generally feasible to evaluate only a small number of samples during a surgical procedure. Infiltrating gliomas are relatively common and have high morbidity and mortality 4. These cancers are defined by the cell-by-cell invasion of surrounding normal brain tissue in potentially any direction from the main tumor mass, but only a minute fraction of the possible areas of infiltration can realistically be examined intraoperatively with current approaches, making undersampling particularly marked.

To improve intraoperative assessment, various optical techniques have been developed for rapid, high-resolution imaging of fresh human brain tissues, including microscopy with ultraviolet surface excitation (MUSE) 5, light-sheet microscopy (LSM) 6, optical coherence tomography (OCT) 7, stimulated Raman scattering (SRS) microscopy 8, 9, and multiphoton microscopy (MPM) 10-15. For MUSE and LSM, fluorescent dyes are required and tissue must be rinsed prior to imaging. Label-free imaging techniques such as OCT, SRS, and MPM are gaining acceptance because of the ease and speed of specimen preparation. Quantitative OCT has successfully demonstrated brain cancer boundary detection based on optical attenuation coefficient mapping 7. However, OCT is not typically designed to achieve subcellular resolution, and has limited biochemical contrast. SRS has shown great promise in detecting brain cancer in fresh human tissues 8, 9, but typically has considerably lower signal-to-noise ratio and speed when performed using the reflection (“epi-”) mode. The transmission mode requires tissue dissection and physical sectioning and is not suitable for *in vivo* imaging.

MPM is a non-destructive technique, capable of high-speed imaging of biological tissues* in vivo* with subcellular resolution 16, 17. Label-free imaging with MPM is possible by capturing two-photon excited fluorescence (TPF) signals from intrinsic fluorophores such as flavin adenine dinucleotide (FAD) and reduced nicotinamide adenine dinucleotide (NADH) 18 and second-harmonic generation (SHG) signals from collagen fibers 16, 17. Previous studies have demonstrated that multiphoton excitation of autofluorescence holds a potential to distinguish cancerous and normal tissue based on fluorescence intensity 19-21. With TPF and SHG signals, MPM is able to image intact bulk tissues in the epi-mode 22 and generate histopathology-like images 23. Furthermore, MPM permits the visualization of unstained and unprocessed tissues within seconds over a large area, and evaluation of successive depths in real time. Although MPM is also subjective to limited sampling, more samples could be assessed in a short period of time to potentially reduce the sampling errors.

This study reports the use of three-channel MPM to evaluate unprocessed and unlabeled human brain tissues from 19 patients. Imaging results were co-registered and compared with clinical standard histopathology. MPM captured several essential diagnostic features and deep learning algorithms applied to MPM images were able to discriminate tissue types. We also demonstrate subcellular resolution label-free imaging of intact brain tissue with a miniature fiber-optic scanning multiphoton endomicroscope that is suitable for *in situ,* high-quality TPF imaging of human tissues intraoperatively.

## Methods

### Sample preparation

Human glioma specimens were obtained from patients undergoing brain surgery at the Johns Hopkins Hospital with patient consent. Normal human brain tissues were obtained from short post-mortem interval autopsies performed on de-identified individuals free of neurologic disease whose next-of-kin consented to research use of tissue. All aspects of the project were performed according to protocols approved by the Institutional Review Board of the Johns Hopkins Hospital.

13 fresh non-cancerous specimens from 8 autopsy cases and 25 fresh specimens from 11 neoplastic neurosurgery cases were included in the study (**Table S1**). Freshly resected specimens were kept in ice-cold phosphate buffered saline solution (PBS) during tissue transportation as well as the entire imaging procedure. The tissue surface was cut flat and covered with a cover glass during imaging. All the measurements were performed in a temperature-controlled (20 °C) dark chamber. After imaging, a subset of specimens were fixed with 10% neutral buffered formalin, and then embedded in paraffin, sectioned at 5 microns, and stained with hematoxylin and eosin.

In addition to label-free MPM imaging, 7 fresh brain specimens including 4 non-cancerous and 3 cancerous cases were stained with a DNA dye, as indicated in the figure captions, either with 5 µM DRAQ5 (BioLengend, Inc.) for 20 min or with 1 µM 4',6-diamidino-2-phenylindole (DAPI) for 2 min, rinsed in PBS for 1 min, and then imaged with MPM again to further assess cytoarchitecture co-registered with the label-free images for comparison and confirmation. The detailed procedure for tissue preparation and imaging is shown in **Figure S1**.

### Multiphoton microscopy setup

**Figure S2** shows a schematic of the MPM imaging system. The custom-built three-channel multiphoton microscopy system was based on an Olympus microscope body (BX61WI, Olympus, Tokyo, Japan). A tunable Ti:sapphire laser (<150 fs, Chameleon Ultra II, Coherent, Inc., CA, USA) was used as the excitation source. The raster-scanned laser beam was focused on the sample by a high numerical aperture water immersion microscope objective (XLUMPlanFl 20x 0.95NA, Olympus, Tokyo, Japan). Epi-fluorescence and SHG signals were collected by the same objective lens and separated from the excitation light with a dichroic mirror, and then further filtered for each of the three channels by a 496 nm long-pass optical filter (F1) for FAD, a 447/60 nm band-pass optical filter (F2) for NADH, and a 390/18 nm band-pass optical filter (F3) for SHG signals before finally coupled into a photomultiplier tube (PMT). Gains and thresholds were set identically for all three PMTs. Images were acquired using an excitation wavelength at 780 nm with an average power of ~30 mW. Images of a size 350 μm × 350 μm from the three channels were collected in a raster fashion, and it took about 9.59 s to acquire one frame of 1024 × 1024 pixels with a 350 μm × 350 μm field of view (FOV). Multiple MPM images were acquired sequentially and automatically stitched to create a single mosaic image with a larger FOV. Three dimensional volumetric images were acquired by automatically scanning the objective focus along depth.

### Multiphoton endomicroscopy setup

The design details of the multiphoton endomicroscope can be found elsewhere 17. Briefly, the multiphoton endomicroscope was built with a custom double-clad fiber (DCF) cantilevered at the distal end of a piezoelectric tube (PZT) with two pairs of orthogonal drive electrodes. The DCF was used for both femtosecond excitation light delivery to and TPF signal collection from the sample. The PZT resonantly scans the fiber cantilever with a spiral pattern by driving the two pairs of electrodes with two amplitude-modulated sinusoidal waveforms that are 90° out of phase. The excitation laser from a tunable Ti:sapphire laser (<150 fs, Chameleon Ultra II, Coherent, Inc., CA, USA) was delivered to the sample through the core of the DCF in the endomicroscope. A grating pair and a dual-fiber scheme were used to manage the temporal pulse properties 24. Fluorescence signals were epi-collected through an achromatic miniature objective into the inner clad of the DCF, and then guided back to the proximal end of the endomicroscope, where they were first separated from the excitation light by a dichroic mirror, and then further filtered for each of the two channels with a 496 nm long-pass optical filter for the FAD and a 447/60 nm band-pass optical filter for the NADH signals before finally being collected by a PMT. The images were taken with the excitation wavelength at 780 nm and an average incident power approximately 40 mW. The FOV of the endomicroscope was around 140 µm in diameter. The use of a single DCF along with the miniature PZT and objective helps reduce the overall probe size. The diameter of the endomicroscope was ~2.8 mm. The small probe size is critical for intra-operative applications to avoid blocking the surgical field of view and ease the delivery of the endomicroscope to the surgical cavity of deep lesions.

### Imaging processing

Automated stitching was performed using custom code written in MATLAB^®^. Quantitative image processing and analyses were performed using ImageJ.

### Machine learning-based classification

A residual convolutional neural network (ResNet) was used for real-time, automated and robust classification of MPM images of unstained human brain tissues 25. A ResNet-34 model was built, trained and validated using Python under the framework of PyTorch.

To test the diagnostic accuracy of the ResNet-based method, 35 specimens from 18 cases were selected by a neuropathologist (Nauen) to be included for the training and validation of two ResNets with ResNet 1 for differentiating normal gray matter and white matter and ResNet 2 for differentiating cancerous and non-cancerous tissues. All the training, validation and testing used each individual images of a size of 350 μm × 350 µm (a single unstitched MPM FOV) within a mosaic. Image tiles within a mosaic that did not contain tissue were excluded. ResNet 1 contained 2,389 MPM FOVs classified either as gray matter or white matter. ResNet 2 contained 3,909 MPM FOVs classified either as cancerous or non-cancerous tissues. For ResNet 1, all FOVs (totaling 2,242 images) from 7 non-cancerous cases (Nos. 1-7) were used for training and validation, and the FOVs (totaling 147 images) from case No. 8 served as the test set. For ResNet 2, all FOVs (totaling 2,494 images) from 12 cases (Nos. 1-6, 9-11, 15-17) were used for training and validation, and the FOVs (totaling 1,415 images) from cases Nos. 7-8, 12-13 and 18-19 served as the test set. Specimen classification was performed at the mosaic level and the specimen was classified as cancer when greater than 50 % of the FOVs within the mosaic were predicted as cancer by the trained neural network.

### *In vivo* multiphoton imaging of mice implanted with human GBM cell lines

In order to test the capacity of MPM for *in vivo* detection of cancer, two 8-week-old immunodeficient male BALB/c nude mice (Charles River Laboratories, Inc., MA, USA) were stereotactically inoculated with cancer cells as previously described 26. Briefly, the mice were injected with approximately 10^6^ GFP labeled GBM1A cells cultured from a patient-derived primary stem cell line 27, which recapitulates the migratory and invasive behavior of human glioblastoma. Mouse brain cancer model and imaging protocols were approved by the Animal Care and Use Committee of Johns Hopkins University.

Four weeks after cancer cell inoculation, the mice underwent MPM imaging. The mice were anesthetized by intraperitoneal injection of ketamine (100 mg kg^-1^ body weight) and xylazine (10 mg kg^-1^ body weight) and maintained by continuous inhalation of isoflurane-oxygen mixture (1-2%). GFP imaging was performed to confirm the development of cancer. Two contralateral burr-holes were made using a dental drill to allow for MPM imaging.

### Imaging system for GFP

An electron-multiplying CCD (EMCCD), equipped with a zoom lens and a long-pass filter (≥496 nm), was used to image the GFP signal from the mouse brain cancer model. A mercury lamp together with a 485/20 nm band-pass filter was employed to provide excitation light.

## Results

### Multichannel label-free brain tissue imaging with MPM

**Figure 1:**
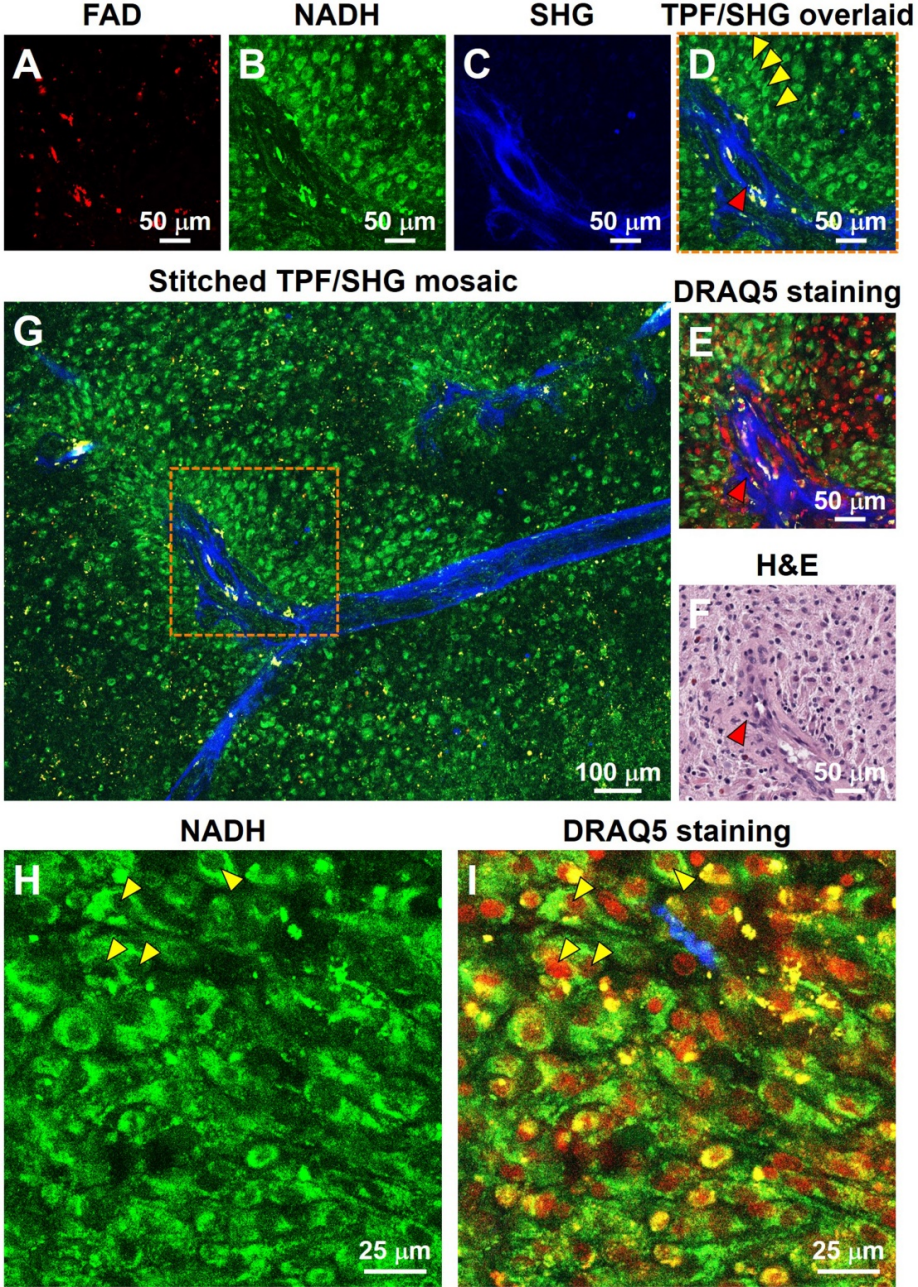
Label-free optical histopathology of freshly excised glioma tissue with MPM 'epi-mode' detection. (**A**) FAD TPF channel, (**B**) NADH TPF channel, (**C**) SHG channel, (**D**) overlaid TPF (FAD & NADH) and SHG image. (**E**) TPF/SHG image after DRAQ5 staining. Label-free TPF/SHG image (**D**) is comparable with the co-located FFPE H&E histology (**F**). (**G**) TPF/SHG mosaic image created by stitching together individual TPF/SHG tiles, with each tile of the size indicate by the dashed square. (**H**) Representative high-magnification NADH TPF image of cancerous tissue with clearly visible nuclear atypia. (**I**) Overlaid TPF/SHG image after DRAQ5 staining to confirm nuclear identity. Red arrowheads: blood vessel; Yellow arrowheads: cancer cell nuclei. Case 18.

For the initial characterization of the ability of our system to resolve subcellular features critical for histopathologic interpretation, we assessed fresh unstained tissue from high-grade glioma (**Figure 1**). Evaluating two-photon fluorescence (TPF) and second harmonic generation (SHG) images of such tissue collected with the MPM system, we found that cellular and subcellular structures are clearly visible in the FAD and NADH channels (**Figure 1A**, **B**), and strong SHG signals from blood vessel walls and collagen deposition (**Figure 1C**) are evident. After acquisition of label-free MPM images, the tissue was labeled with DNA dye DRAQ5 for comparison. The same structural landmarks (e.g., the blood vessel) are found in the label-free image (**Figure 1D**), the DRAQ5-stained image (**Figure 1E**), and the co-located conventional formalin-fixed, paraffin-embedded (FFPE) hematoxylin and eosin (H&E) image (**Figure 1F**). The dark character of nuclei on MPM was seen in all brain regions examined. Nuclear detail in the NADH channel is seen more clearly at higher magnification (**Figure 1H**), in which the dark nuclei correspond exactly to the red cell nuclei in the overlaid DRAQ5-stained image (**Figure 1I**). The dark character of nuclei on label-free MPM images is presumably due to absence of FAD and NADH in this structure. TPF/SHG mosaic images can be created by automated stitching of individual TPF/SHG tiles to obtain a larger field of view (**Figure 1G**). Features critical for pathologic evaluation such as nuclear size and nuclear morphology are readily appreciated in fresh and unlabeled brain tissue viewed with MPM.

### MPM imaging to characterize non-cancerous brain cytoarchitecture

**Figure 2:**
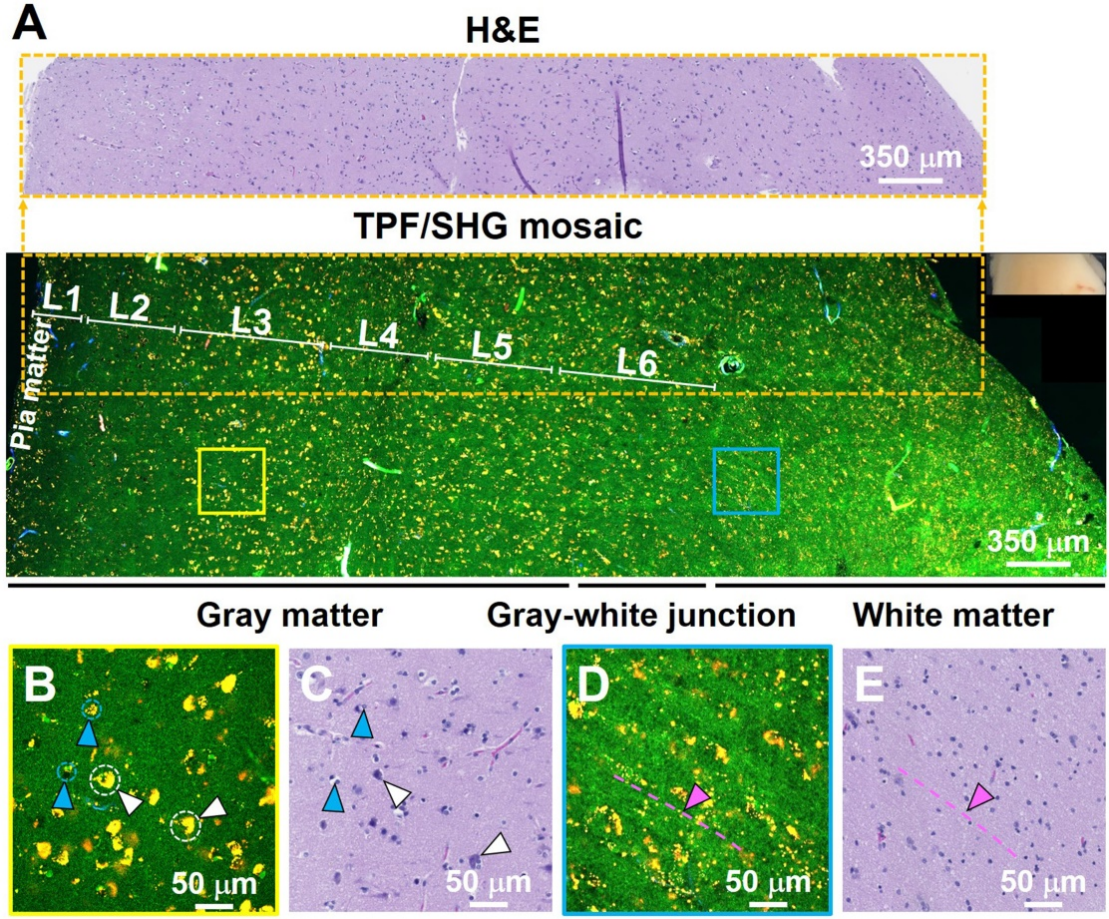
TPF/SHG images of freshly excised unstained non-cancerous cerebral cortex where individual cortical layers can be resolved. (**A**) Mosaic image of the neocortex and subcortical white matter, and co-located H&E histology in the dotted line rectangle. Neurons can be seen at this magnification by their accumulated lipofuscin-like material, visible as yellow granules in our images, and these clusters are proportional to neuron size. Larger clusters can be seen in layers 3, 5, and 6. The smaller neuron size in layers 2 and 4 can also be appreciated. Higher magnification images of (**B**) gray matter; (**D**) subcortical white matter, and (**C, E**) roughly co-located H&E micrographs. Axons are visible in **(D)** as roughly linear structures extending from upper left to lower right, parallel to the dashed line. L1: molecular layer; L2: outer granular layer; L3: outer pyramidal layer; L4: inner granular layer; L5: inner pyramidal layer; L6: pleomorphic layer. White arrowheads: neurons; Light blue arrowheads: glial cells; Pink arrowheads and dashed line: axons. Case 1.

To further test the ability of the system to characterize brain structure cell-by-cell, we next assessed freshly excised unstained human non-cancerous frontal cortex from autopsy. **Figure 2** shows a representative large-scale TPF/SHG mosaic image (5.95 × 1.75 mm^2^) of the cortex with a transition from gray matter (left) to white matter (right). The two regions are readily distinguished by the presence (in gray matter) or absence (in white matter) of larger cells (neurons). The human neocortex is conventionally divided into six layers 28. These layers - molecular, outer granular, outer pyramidal, inner granular, inner pyramidal, and pleomorphic layers - are roughly distinguishable in MPM data (**Figure 2A**; representative higher-magnification images from each layer are shown in **Figure S3**). Moreover, the areas within the dotted box highlight that microstructural features of each layer in the MPM image are in good agreement with those in the corresponding co-located FFPE H&E micrograph. Each layer consists of a characteristic distribution of neuronal sizes, shapes, and densities. The large nuclei, often surrounded by yellow granules, are identifiable as neurons (white arrowheads, **Figure 2B**, **Figure S4**). The granules in the neuronal somata appear to be similar to lipofuscin, emitting in the NADH and FAD channels and thus appearing yellow 29. Smaller dark regions surrounded by few if any fluorescent granules can be identified morphologically as the nuclei of glial cells (light blue arrowheads, **Figure 2B**, **Figure S4**). Compared with gray matter, subcortical white matter (**Figure 2D**, **E**) reveals distinct histologic features, with dense regions of axon matrix (indicated by pink arrowhead and dotted dashed line) that appear as green, roughly linear structures, and interspersed small yellow lipofuscin-like granules, which may arise from myelin membrane remnants 30. Furthermore, fresh human non-cancerous cerebellar cortex was also imaged, and molecular, Purkinje cell and granule cell layers could be clearly identified (**Figure S5**). The results show that MPM is able to provide detailed visualization of the microstructure of human brain.

### MPM imaging of human glioma tissue

**Figure 3:**
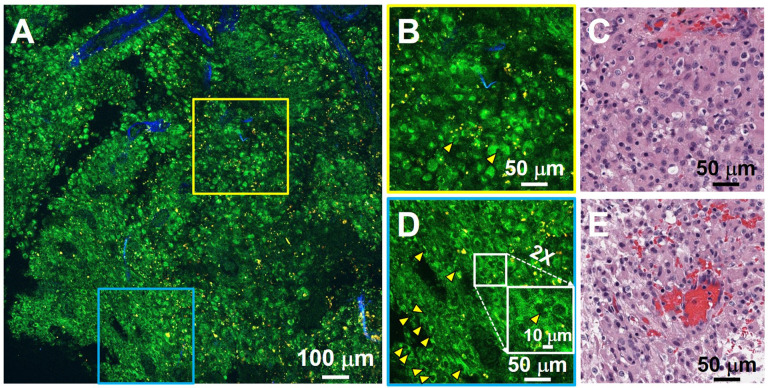
TPF/SHG image of freshly excised unstained high-grade glioma tissue captures hypercellularity and nuclear pleomorphism. (**A**) Mosaic image. (**B, D**) High-magnification TPF/SHG images and (**C, E**) roughly co-located H&E micrographs. The enlarged or irregular nuclei of the glioma are readily visualized (yellow arrowheads). The inset in (**D**) illustrates a typical binucleated cancer cell. Case 18.

For brain cancers, the World Health Organization (WHO) classification scheme is most commonly used 31. Increased cellularity (hypercellularity) can be an important hallmark for brain cancer identification and assessment. Nuclear pleomorphism is another important hallmark used for differentiating cancerous tissue from non-cancerous tissue. **Figure 3** shows an example of unfixed, unlabeled tissue TPF/SHG images and co-located H&E histology obtained from a high-grade glioma. MPM images clearly demonstrate the increased cellularity, showing densely packed individual cancer cells. Cells with an enlarged or irregular nucleus can be identified as cancer cells (yellow arrowheads, **Figure 3B**, **D**). The zoomed-in view (inset in **Figure 3D**) highlights pleomorphism in the form of irregular, enlarged nuclei. The ability to assess the morphology of individual cells is critical for histology-caliber assessment of the presence or absence of infiltrating glioma in a given region.

In additional to auto-fluorescent NADH and FAD, aged brain tissues contain the 'aging pigment' lipofuscin 18, 32. It accumulates in grain-like structures (lysosomes) throughout life 32, and its fluorescence has a punctate appearance. It mainly accumulates in neurons and microglia 32. Furthermore, myelin fragments are frequently associated with lipofuscin, suggesting that some of the lipofuscin may arise from myelin membrane remnants 30. Lipofuscin can be excited by near-infrared light (760 to 980 nm) 32, and has a broad emission spectrum ranging from 480 to 700 nm 18, 32. In the non-cancerous cerebral cortex under MPM imaging, lipofuscin-like granules are readily observed in neurons. Abundant small fluorescent granules are also found in non-cancerous white matter. As lipofuscin may accumulate with age, tissue from younger brains has far fewer yellow lipofuscin-like signals than older brain tissue (**Figure S6**). Moreover, compared with non-cancerous tissues (**Figure 2B, D**), far fewer yellow lipofuscin-like granules are observed in the cancerous tissue, which may reflect less accumulation of lipofuscin in the newly grown brain cancer cells. This distinction is less readily seen in conventional FFPE H&E micrographs and could provide additional diagnostic information for visual assessment by expert histopathologists.

**Figure 4:**
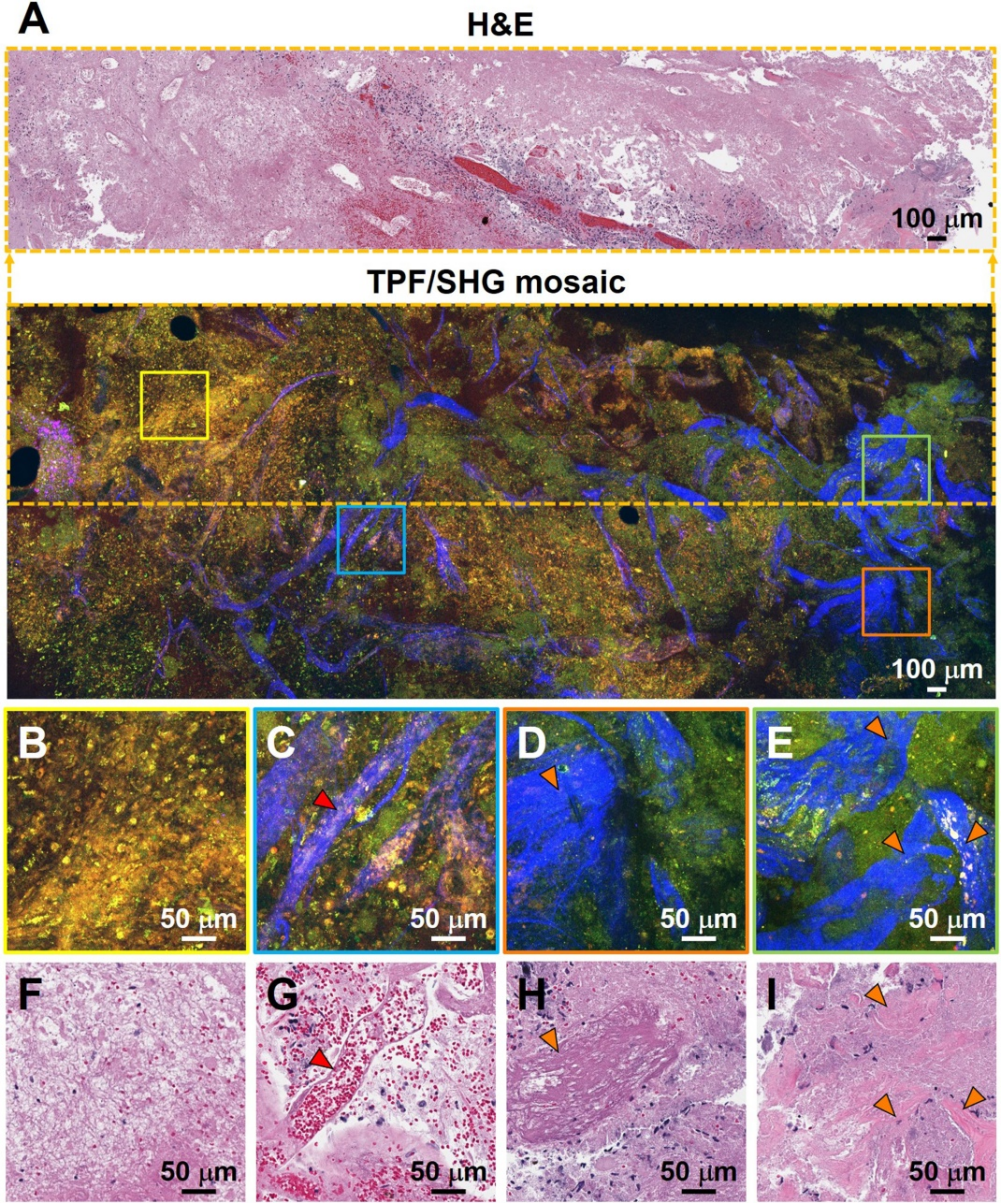
Characterization of necrosis, microvascular proliferation, and collagen deposition using TPF/SHG of unlabeled fresh high-grade glioma tissue. (**A**) Mosaic image of high-grade glioma and roughly co-located H&E histology in the dotted line rectangle. Higher magnification images of (**B**) region of necrosis; (**C**) extensive microvascular proliferation; (**D**) thrombosed vessel; (**E**) direct collagen deposition, and (**F-I**) roughly co-located H&E micrographs. Red arrowheads: blood vessel; orange arrowheads: collagen deposition. Case 17.

Additional hallmarks of glioblastoma are the presence of tortuous and disorganized blood vessels (termed microvascular proliferation) 33, 34, necrosis, and in some cases extracellular collagen deposition. SHG signals from the collagen of the vessel walls has demonstrated significantly increased vascularization in cancers 34. **Figure 4** details TPF/SHG images of the appearance of vascularization along with necrosis and collagen deposition in freshly excised unstained high-grade glioma tissue. In the necrotic area (**Figure 4B**), cell outlines are still visible ('coagulative necrosis'), and nuclei appear smaller or absent, similar to their appearance on H&E. Collagen in the adventitia of larger blood vessels in the high-grade glioma is well visualized (**Figure 4C**). **Figure 4D** and** 4E** highlight collagen deposition in the extracellular matrix, which show dense and clustered blue SHG signals over a larger area. By highlighting collagen either from the basement membrane of blood vessels or from direct deposition, and demonstrating changes in necrotic cells, MPM is well suited for revealing diagnostic features of glioblastoma. **Table S2** comparatively summarizes the key diagnostic histologic features of non-cancerous and cancerous brain tissues in the MPM and H&E images.

Moreover, MPM can acquire images at various depths, which is analogous to traditional serial sectioning from a paraffin block. *Ex vivo* this would obviate the need for tissue flatness during imaging of fresh, fixed, or fixed/embedded tissues, and *in vivo* it would permit assessment of multiple depths without moving the probe. The penetration depth with a given wavelength and incident power is highly dependent on the type of tissue, and it was found that a deeper MPM imaging depth could be achieved in non-cancerous gray matter and high-grade glioma tissues than in non-cancerous white matter (**Figure S7**). This result is consistent with our previous finding that glioma tissue has significantly lower optical attenuation when compared with non-cancerous white matter 7.

### Deep learning-based classification

Interpretation of histopathological images require a highly trained pathologist and is often time-intensive, and subject to inter-observer variability. Recently, machine-learning based methods for diagnostic classification have been increasingly applied to microscopy images 8, 9, and the results show promise for applications in automated disease diagnosis. In order to demonstrate a proof-of-concept use of a deep learning model for automated diagnostic predictions of MPM images, we employed ResNet-34 for classification of fresh unstained human brain tissues.

**Figure 5:**
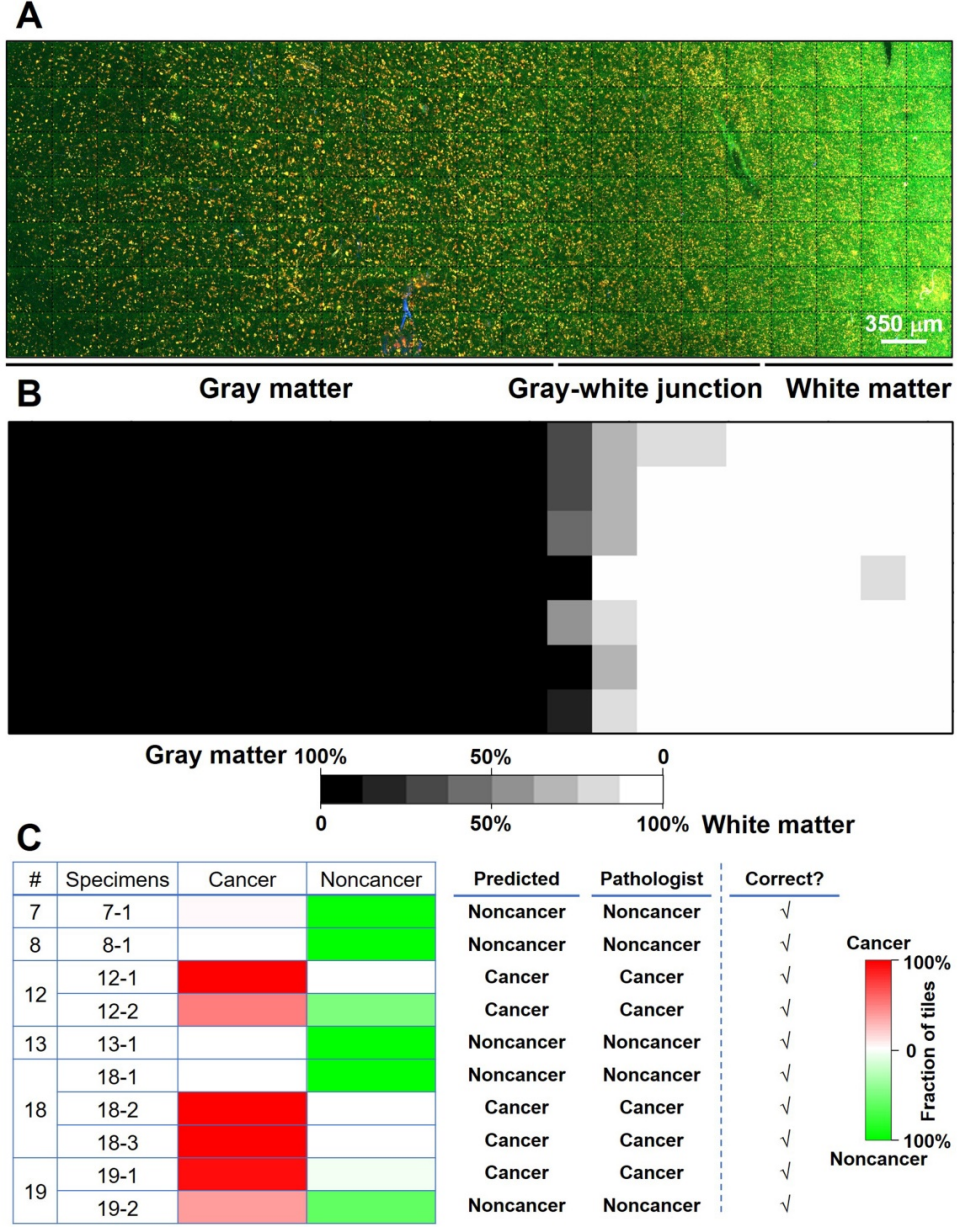
Preliminary deep-learning based classification of MPM images achieves high accuracy. (**A**) MPM mosaic image of a fresh non-cancerous human cortex (Case 8) containing gray matter and white matter. (**B**) Probability heatmap generated by ResNet 1 for each MPM FOV across the entire specimen for predicting whether each FOV is gray or white matter. (**C**) Summary of ResNet-based classification of specimens as cancerous or non-cancerous tissue for a test set of 6 cases (Cases No. 7, 8, 12, 13, 18, and 19) with a total of 10 specimens. Representative images from the 10 specimens are presented in **Figure S8**.

We proposed and trained two ResNet models. The first one (ResNet 1) was trained to distinguish gray matter and white matter. The corresponding H&E histology served as the ground truth. Training, validation, and testing used each individual image (350 × 350 µm^2^, the size of a single unstitched MPM FOV) within a mosaic. This network makes predictions on an individual MPM FOV level. Our result shows that the trained network could predict gray versus white matter at the MPM FOV level with a 94% accuracy. **Figure 5A** shows the mosaic TPF/SHG image of a fresh non-cancerous human cortical specimen including gray matter and white matter. The gray matter, gray-white junction, and white matter can be clearly identified based on the probability heatmap (**Figure 5B**) generated by ResNet 1. The second ResNet (ResNet 2) was trained to distinguish cancerous versus non-cancerous brain tissues. Our result shows that the trained ResNet 2 could make this distinction with a 92% accuracy at the individual MPM FOV level. At the mosaic level of each specimen, classification can be achieved based on the assessment of individual FOVs within the given mosaic. An individual FOV is categorized as cancer when the probability predicted by the trained ResNet 2 is more than 50%. At the mosaic/specimen level, the entire specimen is classified as cancer when greater than 50% of the FOVs within the mosaic are classed as cancer. As an example, ResNet 2 correctly assessed 154 out of 163 MPM FOVs from specimen #19-1 as cancer, and the remaining 9 FOVs as normal, so the specimen is classified as cancer. By using this approach, classification of MPM mosaic images at the specimen level as cancerous or non-cancerous achieved 100% accuracy (**Figure 5C**).

### Multiphoton endomicroscopy of human brain tissues

**Figure 6:**
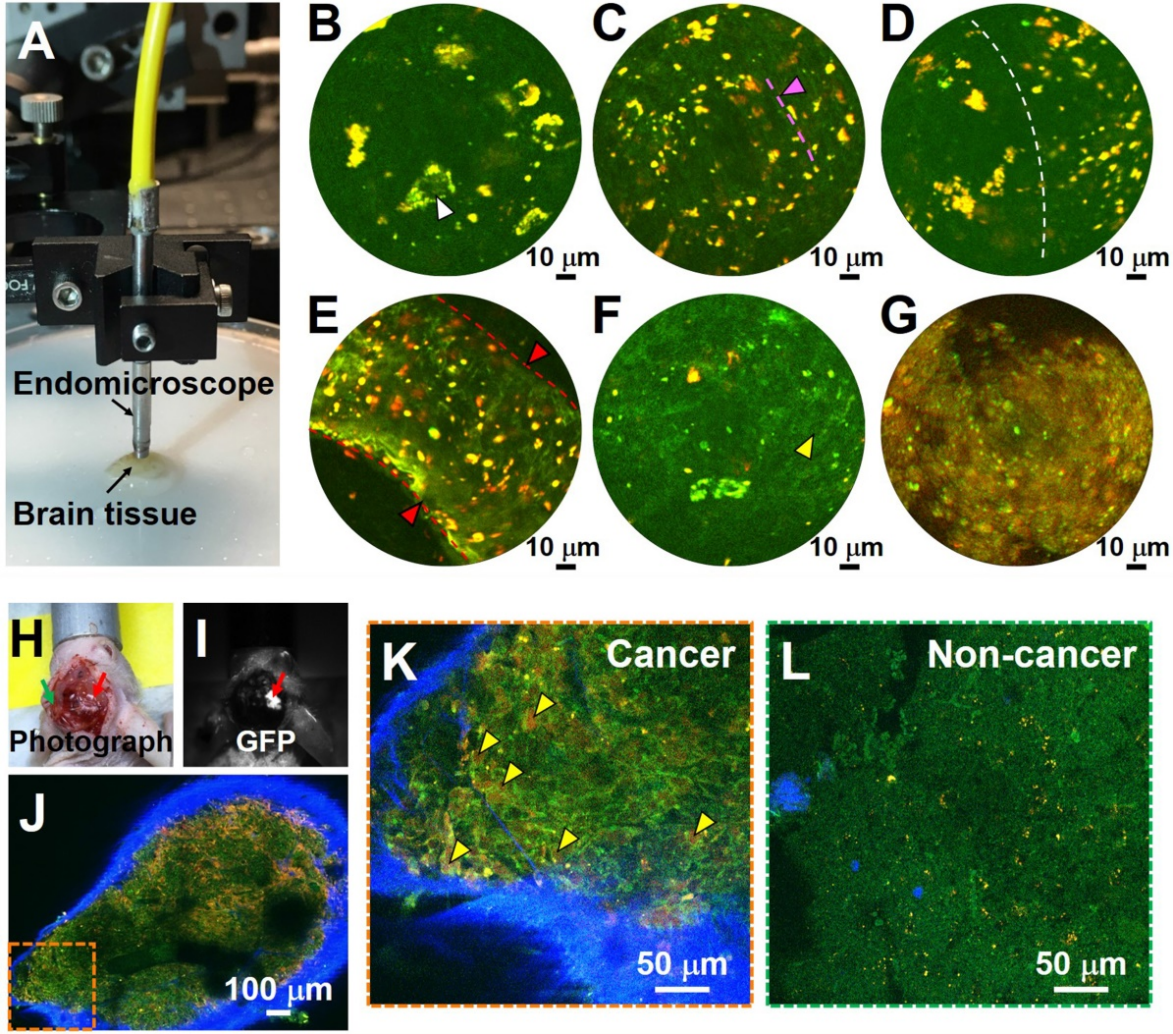
Label-free endomicroscopic TPF imaging of freshly excised non-cancerous (Case 7) and cancerous human brain tissues (Cases 17 and 19), and *in vivo* detection of cancer in a patient-derived mouse brain cancer model with MPM. (**A**) Photo of the multiphoton endomicroscope over a human brain specimen. (**B**) Non-cancerous gray matter shows scattered pyramidal neurons (as indicated by white arrowhead) with angulated boundaries and yellow lipofuscin-like granules. (**C**) Non-cancerous white matter. The green roughly linear structures (as indicated by pink arrowhead and dashed line) are axons. (**D**) Border between non-cancerous gray and white matter. (**E**, **F**) TPF images of high-grade glioma. (**G**) Necrotic area in high grade glioma shows loss of cellular structure. SHG signal was not used for the endoscopic images. (**H**) Photograph of patient-derived mouse brain cancer model with two contralateral burr-holes. Red arrow indicates the burr-hole in the cancer area and the green arrow indicates the burr-hole in the contralateral non-cancer area. (**I**) Corresponding image in GFP channel. Red arrow indicates the cancer area with bright GFP signal. (**J**) Mosaic TPF/SHG images of cancer area *in vivo*. High-magnification TPF/SHG image of (**K**) cancer and (**L**) contralateral non-cancer area in mouse brain *in vivo*, with many morphologically normal darker regions corresponding to nuclei. Yellow arrowhead: enlarged cancer cell nucleus; red arrowhead and dashed line: blood vessel.

The results described above demonstrate that MPM can be used for label-free, non-destructive and rapid imaging of human brain tissues with subcellular histological detail. The approach is thus suitable to guide the neurosurgeon intraoperatively and in real time by providing neuropathological information on the surgical 'margin' to safely maximize glioma resection while sparing adjacent heathy brain tissue. Our group previously reported an ultracompact, fiber-optic nonlinear endomicroscope for label-free functional histological imaging *in vivo* 17*.* Here, we further assess the ability of the multiphoton endomicroscope to image fresh and unlabeled human brain tissue. Multiphoton endomicroscope-obtained images of unfixed non-cancerous and cancerous human brain tissues are provided in **Figure 6**. **Figure 6A** shows a photo of the endomicroscope with a diameter of ~2.8 mm. The laser power delivered to the sample was ~40 mW and the imaging speed was ~2 frames/sec. These images were averaged over twenty frames. We observed large neurons (white arrowheads) with dark nuclei and angular cell bodies containing lipofuscin-like granules in non-cancerous gray matter (**Figure 6B**). White matter axons appear as green nearly linear structures (indicated by pink arrowhead and dashed line) and smaller fluorescent deposits are found in the non-cancerous white matter (**Figure 6C**). **Figure 6D** shows a clear border between white and gray matter (indicated by the dashed line). Large blood vessels (red arrowheads) and cancer cells (yellow arrowheads) with dark nuclei and fewer yellow lipofuscin-like granules can be found in high-grade glioma tissue (**Figure 6E**, **F**). Cell outlines are still visible, and nuclei appear absent in the necrotic area of high-grade glioma (**Figure 6G**). Although the images presented in **Figure 6** are averaged over twenty frames, the corresponding single-frame images (**Figure S9**) capture similar structural details, demonstrating the excellent detection sensitivity of the multiphoton endomicroscope. Use of *ex vivo* samples is critical for development of the system. For a proof-of-concept demonstration, we conducted *in vivo* MPM imaging on a mouse model implanted with GFP-labeled cells from GBM1A, a patient-derived cell line 26, 27. As shown in **Figure 6H-L**, MPM permits *in vivo* identification of glioma cells with enlarged nuclei (**Figure 6K**), which were confirmed by GFP fluorescence (**Figure 6I**) to be cancerous.

## Discussion

The most commonly used method to generate diagnostic quality tissue sections is fixation in formalin, followed by paraffin infiltration, sectioning, and staining. Since this tissue preparation process requires at a minimum many hours and often in practice several days, alternative histology techniques including frozen sectioning and smear preparations are used when intraoperative assessment is required. These methods are far faster, requiring only a few tens of minutes from the time of tissue removal to viewability on the microscope. However a) the fastest turnaround time for a tissue sample to be taken from the operating room to the histology laboratory for freezing, cutting, staining and assessment is approximately 10 minutes under optimal conditions, which is a lengthy delay for clinical decision making during a surgical procedure, b) the freezing process may introduce geometric distortion, causing significant image artifacts 3, and the smear preparation, while preserving cytologic detail, essentially abolishes architectural information, c) only a very limited proportion of possibly involved tissue can be assessed by this method, and d) tissue must be first be excised from the brain for assessment, despite the possibility that it may be normal. In comparison, MPM can directly image intact human brain tissue. This technique can eliminate time-consuming tissue processing, and thus enables real-time assessment of the pathology. With the development of the endomicroscope, *in vivo* imaging can be possible without need for tissue removal, sectioning or processing. Currently, the frame rate of the endomicroscope is ~2 frames/sec, which continues to improve, permitting rapid assessment 35.

Our study demonstrates that MPM is able to capture essential diagnostic hallmarks for brain cancer identification and classification. Previous studies have demonstrated that that multiphoton excitation of autofluorescence hold the potential to distinguish tumor tissue and normal brain based on the intensity and lifetime of fluorescence 10, 11. Some microenvironment characteristics in gliomas, such as collagen deposition in extracellular matrix and tumor angiogenesis, were identified in the MPM images for the brain slices after frozen cutting previously 14, 15. We found that MPM provides structural information similar to that of the co-located histopathology for fresh brain tissues. We have demonstrated that MPM robustly captures diagnostic features based the structural information in the MPM images, which permits near real-time intraoperative optical histopathology. The number, size, distribution, and morphology of cell bodies, nuclei, and blood vessels within a given brain region can be determined, which are important indicators to determine whether a lesion is present and, if so, to characterize the lesion. Furthermore, MPM allows detection of collagen which can be difficult to distinguish by conventional methods without special staining. The issue of progression versus radiographic “pseudoprogression” due to tissue changes including necrosis and edema following therapy is of tremendous importance and is a frequent cause of intraoperative consultations to neuropathology. Our ability to assess cellularity and atypia (**Figure 3**) and our clear identification of necrosis (**Figure 4**) will enable the pathologist using MPM to visualize the tissue to effectively distinguish active tumor from treatment effect. Such ability could in principle be used for identification other types of lesions (e.g., inflammation, infarct, demyelination, and other common neuropathologic lesions) or indeed of any microscopic tissue pattern. Although we are optimistic that our resolution (**Figure 1**) will permit identification of mitotic figures, further testing is needed to confirm. Furthermore, with MPM, the operator can view in real time tissue microarchitecture over different regions of interest in three dimensions simply by panning and adjusting the imaging beam focus, which will likely further improve the diagnostic efficiency, sensitivity and accuracy.

NADH and FAD are often sequentially excited at 700-750 nm and 850-920 nm, respectively, in order to obtain cleaner (or less mixed) fluorescence signals. This sequential excitation at different wavelengths, however, would lead to a lower imaging frame rate, and more importantly, it would become more difficult in ensuring pixel-level registration between the NADH and FAD channels in the case of dynamic samples (e.g., when performing imaging *in vivo*) 36, 37. Here we chose 780 nm laser to simultaneously excite and collect the fluorescence of NADH and FAD to avoid the above mentioned difficulties. In the future, spectral unmixing would be explored to minimize the crosstalk between the NADH and FAD channels.

Machine learning-based diagnosis may be able to ultimately reduce inter-observer variability and support real-time standardized intraoperative pathology. We trained and implemented a ResNet model for rapid, automated intraoperative classification of MPM images of fresh and unstained brain tissues. Our preliminary results demonstrated that MPM-based machine-learning methods can be used for discrimination of non-cancerous and cancerous tissues. However, because of the restricted sample size, some MPM FOVs may have unique numbers, sizes, distributions, and morphologies of cell bodies, nuclei, and blood vessels that are beyond the general features included in the training set, and thus the diagnostic accuracy was only 92% at the FOV (350 × 350 µm^2^) level. We hypothesize that the diagnostic accuracy would be improved by increasing the sample size.

Several compact MPM platforms have been recently developed to facilitate the translation of MPM to *in vivo* imaging. A multiphoton microscope with a GRIN-lens based needle-like objective has been developed and employed for *in vivo* glioma tissue imaging during glioblastoma resection 38. However, the size remained too large for intracranial use. Another exciting development is multiphoton endomicroscopy technology, which is positioned for *in vivo* imaging 17, 39. We previously showed the miniature fiber-optic multiphoton endomicroscope that was employed in the present study was capable of label-free *in situ* imaging of various tissues, including mouse liver, small intestine, and cervix, with subcellular histological detail and high image quality 17, 40. Here we demonstrate that the multiphoton endomicroscope is able to image unfixed, unstained human brain tissue with reasonable image quality; we are optimistic that continued technical advances in resolution and field of view will further improve the images. To prepare for intra-operative imaging of human brain *in vivo*, the safe laser irradiation level will have to be further determined, though the incident laser power of 40 mW used in our endomicroscopy imaging has been viewed as safe 41, 42. Tissue can be frozen for use in sequencing or fixed for histological assessment after multiphoton imaging; as shown in **Figures 1** to **4**, no visible damage was observed in the correlated histopathologic images after multiphoton imaging. Nonetheless, systematic animal experiments are still needed to confirm the intraoperative safety and feasibility of *in vivo* multiphoton microscopy imaging for surgical guidance. We note that the same imaging device can be potentially used for laser ablation/coagulation therapy when neoplastic cells are identified with the same imaging laser (but at a higher power) or a dedicated laser operating an efficient ablation wavelength (*i.e.*, 1,470 nm) 43. In addition, multiphoton endomicroscopy can also perform functional histologic imaging *in vivo* to reveal cellular metabolic/redox status given by the ratio of FAD and NADH fluorescence of biological tissue 17, providing novel information to potentially improve detection of glioma infiltration.

Multiphoton endomicroscopy represents a promising pathway for *in vivo* optical histologic imaging of brain cancer. The current FOV diameter is roughly 140 microns and the imaging depth is about 200 microns. As a result, it is not possible to scan a large tissue area/volume with one FOV. For future clinical use, multiphoton endomicroscopy can be robotically co-registered with a surgical microscope or integrated with other wide-field imaging technologies for complementary image guidance over a large area of the surgical cavity. The imaging speed and FOV of the endomicroscopy technology are under active investigation and expected to be improved significantly in the near future to meet needs for performing real-time intra-operative optical histology *in vivo*. Use during surgery in the fashion we envision would permit diagnosis to be integrated more closely with treatment than generally possible. When ultimately combined with MRI-based techniques for localization 44 and OCT-based tools for tissue characterization 7, the availability of subcellular structural information within seconds from regions around a suspected tumor has the potential to markedly improve patient outcomes.

## Supplementary Material

Supplementary figures and tables.

## Abbreviations

DCF: double-clad fiber
EM-CCD: electron-multiplying CCD
FFPE: formalin-fixed, paraffin-embedded
FAD: flavin adenine dinucleotide
FOV: field of view
H&E: hematoxylin and eosin
LSM: light-sheet microscopy
MPM: multiphoton microscopy
MUSE: microscopy with ultraviolet surface excitation
NADH: reduced nicotinamide adenine dinucleotide
OCT: optical coherence tomography
PBS: phosphate buffered saline
PMT: photomultiplier tube
PZT: piezoelectric tube
ResNet: residual convolutional neural network
SRS: stimulated Raman scattering
SHG: second-harmonic generation
TPF: two-photon excited fluorescence
WHO: World Health Organization
