# Supplementary material for: Label-free imaging of human brain tissue at subcellular resolution for potential rapid intra-operative assessment of glioma surgery
Source: Theranostics. 2021 May 24;11(15):7222–34. doi: 10.7150/thno.59244 (PMC8210590; doi:10.7150/thno.59244)
Supplement: Supplementary file 1 — Supplementary figures and tables. [file thnov11p7222s1.pdf]

Supplementary Information for

Label-free imaging of human brain tissue at subcellular resolution for potential rapid intra-operative assessment of glioma surgery

Defu Chen,[1,2,6] David W. Nauen,[3,6] Hyeon-Cheol Park,[1] Dawei Li,[1] Wu Yuan,[1] Ang Li,[1] Honghua Guan,[1] Carmen Kut,[1] Kaisorn L. Chaichana,[4] Chetan Bettegowda,[5] Alfredo Quinones-Hinojosa,[4,*] Xingde Li[1,*]

[1]*Department of Biomedical Engineering, Johns Hopkins University School of Medicine, Baltimore, Maryland 21205, USA*

[2]*Institute of Engineering Medicine, Beijing Institute of Technology, Beijing 100081, China*

[3]*Department of Pathology, Johns Hopkins University School of Medicine, Baltimore, Maryland 21205, USA*

[4]*Deparmtent of Neurologic Surgery, Mayo Clinic, Jacksonville, FL 32224, USA*

[5]*Department of Neurosurgery, Johns Hopkins University School of Medicine, Baltimore, Maryland 21205, USA*

[6]*Equal contribution*

[*]Corresponding author. E-mail: xingde@jhu.edu (X.L.); Quinones-Hinojosa.Alfredo@mayo.edu (A.Q.H.)

SUPPLEMENTAL MATERIALS

Supplementary Figures S1-S9

Fig. S1: Procedure for brain tissue preparation and imaging.
Fig. S2: Schematic of the home-built TPF/SHG multiphoton microscopy system.
Fig. S3: Representative higher-magnification images from each layer in normal cortex.
Fig. S4: TPF/SHG images of freshly excised unstained non-cancerous human gray matter.
Fig. S5: Label-free MPM images of freshly excised unstained non-cancerous cerebellar cortex demonstrate near-histologic level detail.
Fig. S6: TPF/SHG images of freshly excised unstained human brain cortex from different ages.
Fig. S7: MPM permits robust optical sectioning and detects differences along depth among brain tissues.
Fig. S8: Representative images of each of the 10 specimens used for classification testing of deep learning of cancer and non-cancer classification network.
Fig. S9: Single-frame endomicroscopic label-free TPF images of intact human brain tissues.

Supplementary Tables S1-S2

Table S1: Clinical and histopathological characteristics of our human samples.
Table S2: Summary of key diagnostic histologic features of non-cancerous and cancerous brain tissues.

Supplementary Figures

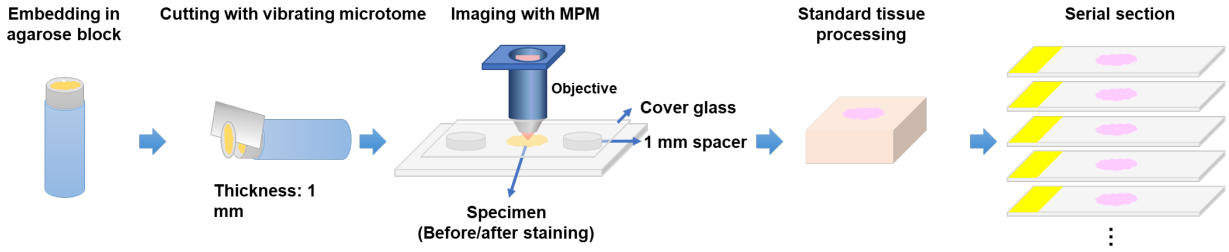

Figure S1 | Procedure for brain tissue preparation and imaging. Freshly excised human brain tissue was put in a cylindrical container and covered with low-melt agarose at 40 ^{o}C and allowed to solidify on ice. The agarose block containing the tissue piece was then sectioned with a vibrating microtome (VF-300, Precisionary Instruments, Inc., Greenville, NC, USA). The thickness was set to 1 mm. Then the specimen was covered with a cover glass during imaging. The cover glass was supported by two spacers (1 mm thickness) to prevent tissue compression. In some cases, tissue was imaged before and after nuclear staining. After imaging, a subset of the specimens were fixed with 10% neutral buffered formalin then processed, embedded in paraffin, sectioned at 5 microns, and stained with hematoxylin and eosin. Serial *en face* sectioning was performed in order to find the best co-registered H&E images.

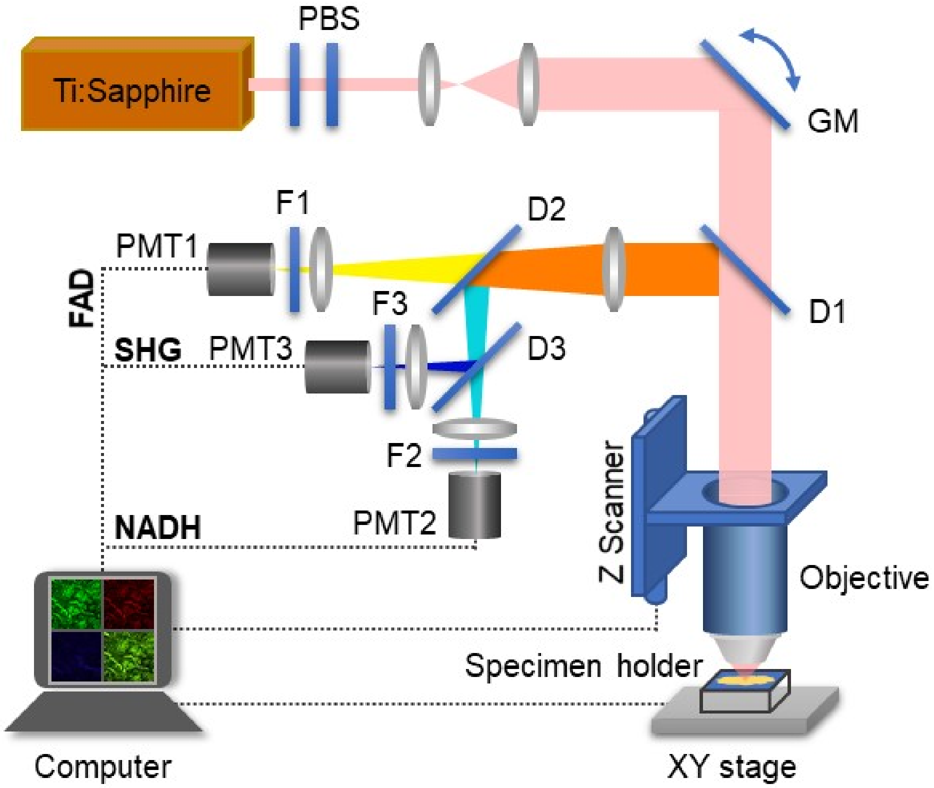

Figure S2 | Schematic of the home-built TPF/SHG multiphoton microscopy system. TPF and SHG signals are epi-collected with three photomultiplier tubes. F1: 496 long-pass optical filter; F2: 447/60 nm band-pass optical filter; F3: 390/18 nm band-pass optical filter; D1: 705 nm long-pass dichroic beam-splitter, D2: 496 nm long-pass dichroic beam-splitter, D3: 405 nm long-pass dichroic beam-splitter; PBS: polarization beam splitter; GM: galvanometer; PMT: photomultiplier tube. Gains and thresholds were set identically for all three PMTs.

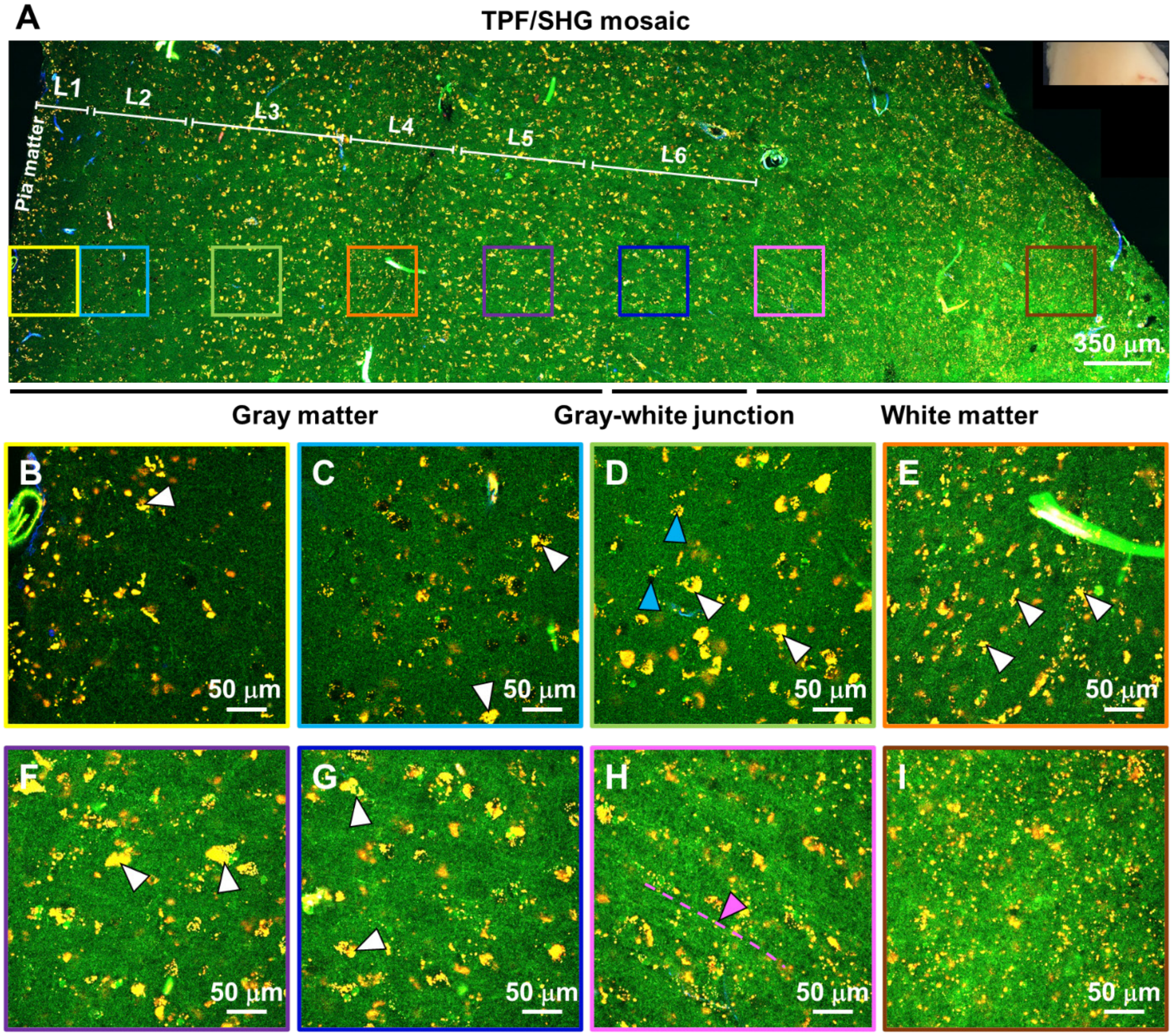

Figure S3 | Representative higher-magnification images from each layer in normal cortex. (A) Mosaic image of the neocortex and subcortical white matter. (B) Molecular layer (L1); (C) Outer granular layer (L2); (D) Outer pyramidal layer (L3); (E) Inner granular layer (L4); (F) Inner pyramidal layer (L5); (G) Pleomorphic layer (L6); (H) Gray-white junction; (I) White matter. Whereas cell nuclei are darker green than cytoplasm and neuropil, foci of edema, prominent in (B) and (C), are devoid of signal and hence completely black. White arrowheads: neurons; Light blue arrowheads: glial cells; Pink arrowheads and dotted dashed line: axons. (Case 1)

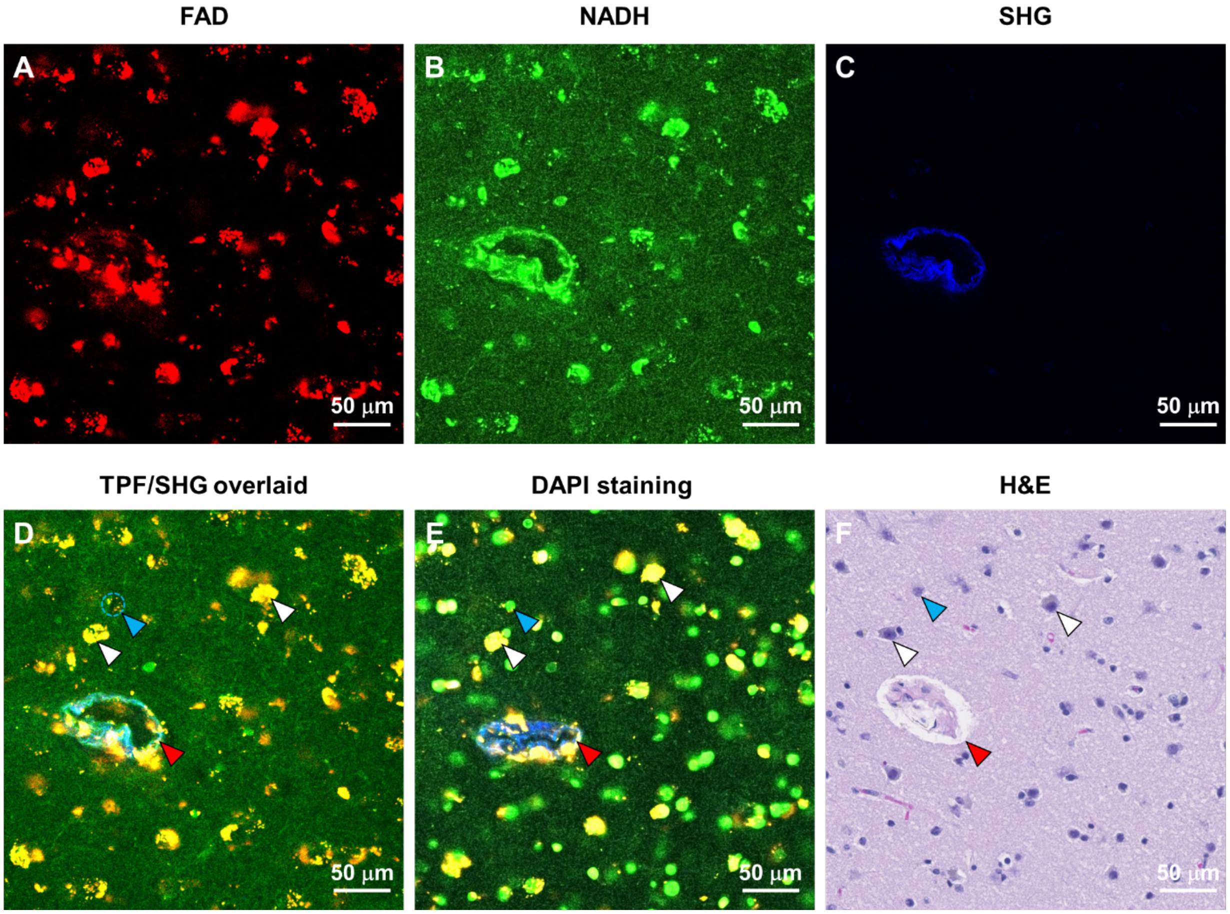

Figure S4 | TPF/SHG images of freshly excised unstained non-cancerous human gray matter. (A) FAD TPF channel, (B) NADH TPF channel, (C) SHG channel, (D) overlaid TPF/SHG image. (E) TPF/SHG image after DAPI staining. TPF/SHG image (D) is comparable with the co-located H&E histology (F). The fluorescence emission peak of DAPI is mainly located in the NADH channel. The same structural landmarks (i.e., the blood vessel as indicated with red arrowheads) can be found in the label-free image (D), the DAPI-stained image (E) and the co-located conventional FFPE H&E image (F), respectively. The clusters of yellow granules in (D) correspond with the green cell nuclei in the DAPI-stained image (E), confirming that the clusters delineate cells. Nuclei appear to harbor little FAD or NADH, and thus appear much darker than the surrounding cytoplasm and neuropil. By comparing panel (D) with panel (F), the clusters of yellow (that is, present on both FAD and NADH channels) spots surrounding dark regions, as indicated with white arrowheads, are identifiable as neurons. Smaller dark regions surrounded by few if any fluorescence granules can be identified morphologically as glial nuclei (light blue arrowheads).

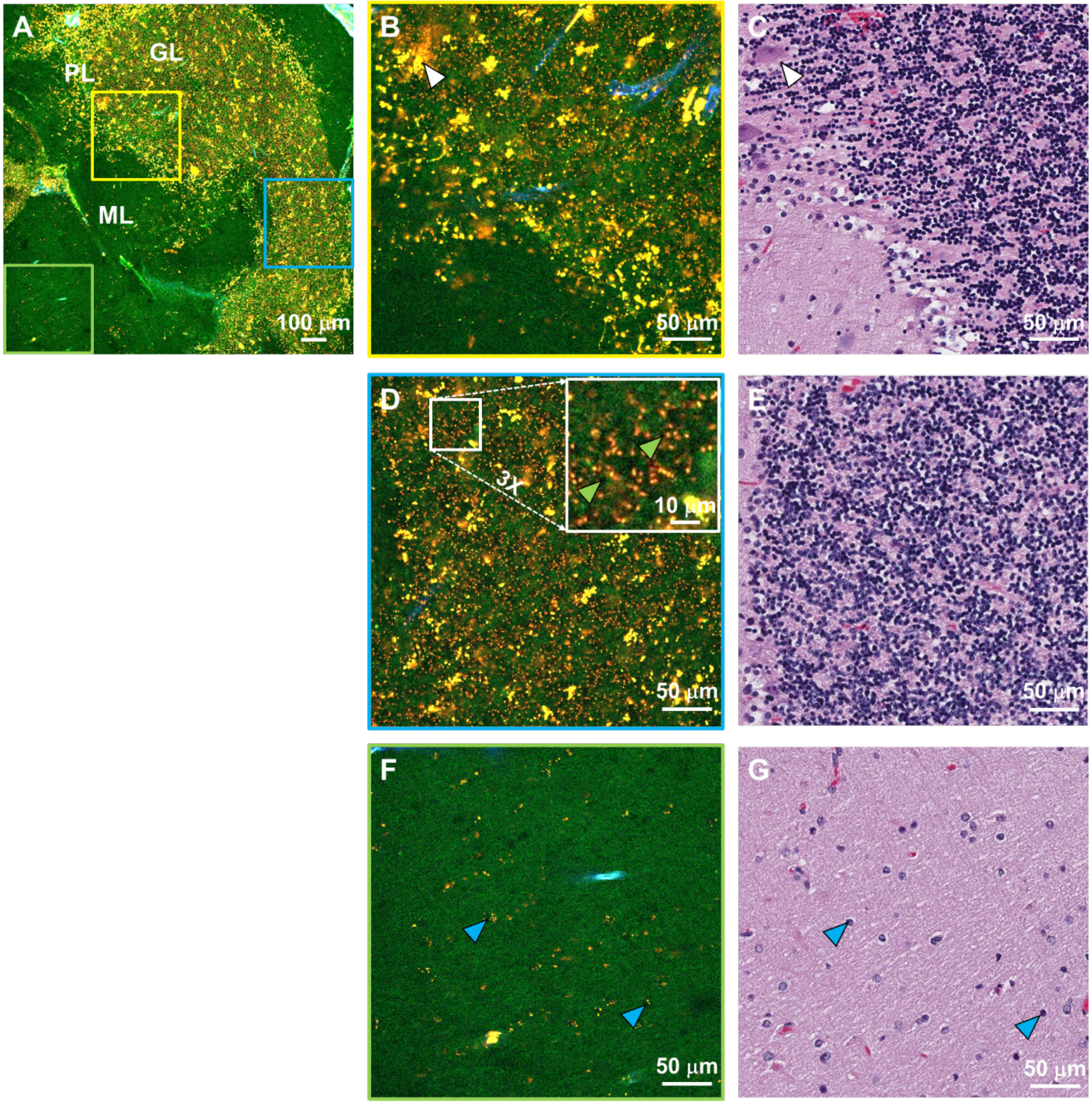

Figure S5 | Label-free MPM images of freshly excised unstained non-cancerous cerebellar cortex demonstrate near-histologic level detail. (A) Mosaic image of cerebellar cortex, (B) Purkinje cell/Bergmann glial cell layer (PL), (D) granule cell layer (GL), (F) molecular layer (ML). (C, E, G) Co-located H&E histology. White arrowheads: Purkinje neurons, light green arrowheads: granule cell nuclei, light blue arrowheads: glial nuclei. The molecular and internal granule cell layers are separated by the Purkinje cell/Bergmann glial cell layer, which appears bright yellow due to the strong TPF signals. The magnified border between layers is shown in Figure (B), the granule cell layer with densely packed nuclei is shown in panel D, and the molecular layer with sparse glial nuclei is shown in Figure (F). As in neocortex, nuclei appear as dark (but not pure black) regions due to lack of fluorescent signal. The tissue architecture in TPF/SHG images correlates well with the co-located FFPE H&E images. (Case 14)

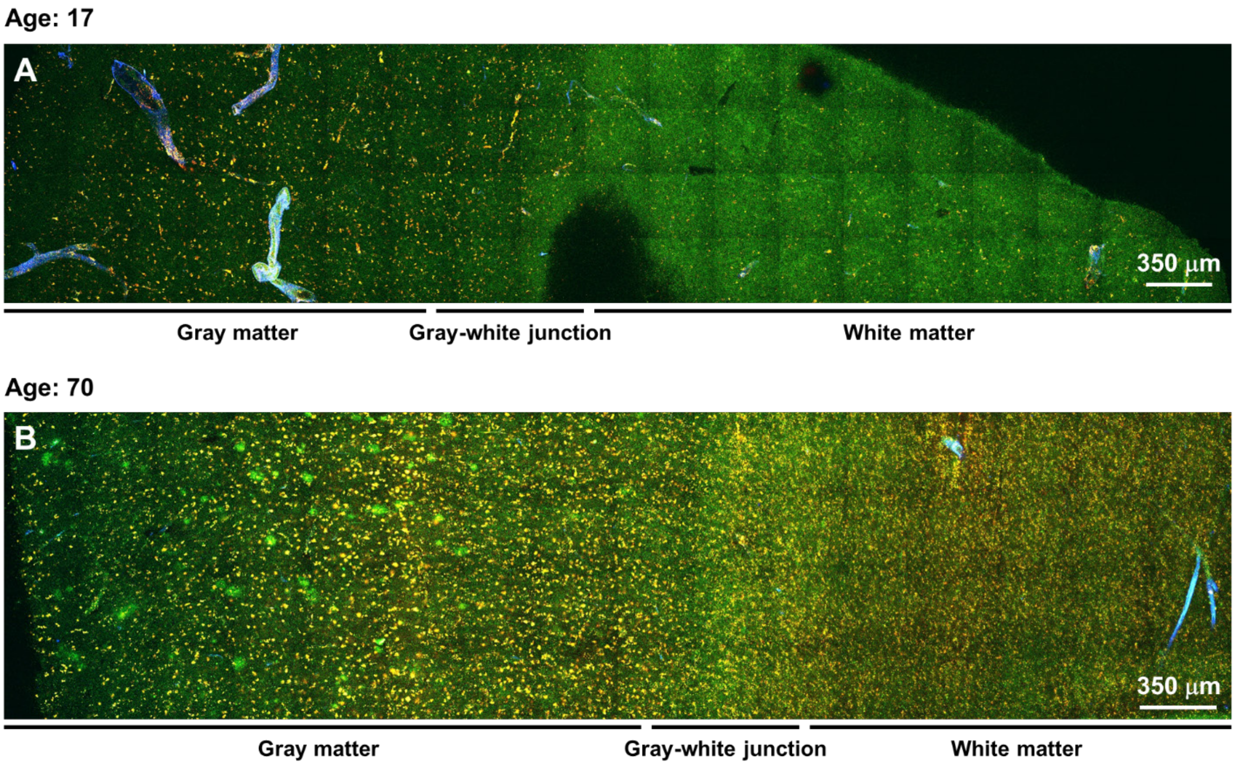

Figure S6 | TPF/SHG images of freshly excised unstained human brain cortex from different ages. (A) Mosaic image from a 17-year-old individual (Case 4). (B) Mosaic image from a 70-year-old individual (case 8). The brain tissue from the younger person (A) exhibits far fewer yellow lipofuscin-like signals compared to that from the elderly person (B). The same incident laser power and detector gain were used for all the images.

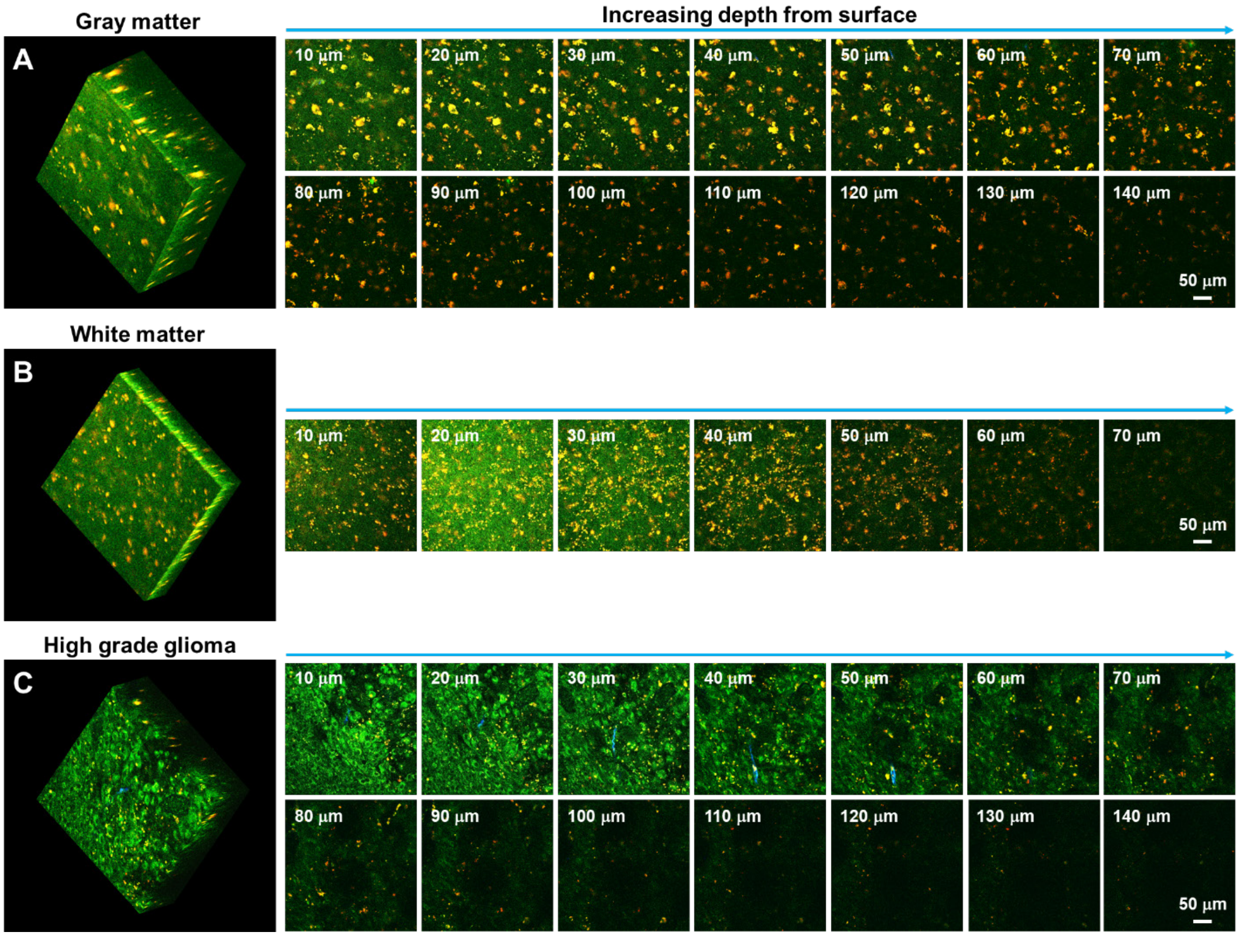

Figure S7 | MPM permits robust optical sectioning and detects differences along depth among brain tissues. (A) Representative TPF/SHG images from non-cancer gray matter (Case 8). (B) Representative TPF/SHG images from non-cancer white matter (Case 8). (C) Representative TPF/SHG images from brain cancer tissue (Case 18). The volumetric reconstruction and individual frames show the image data from the surface to 140 µm deep for gray matter (A), from the surface to 70 µm deep for white matter (B), and from surface to 140 µm deep for brain cancer tissue (C). The same incident laser power and detector gain were used for all the images. For non-cancer white matter, the TPF signal becomes very dim when the imaging depth is more than 70 µm (B). However, for non-cancer gray matter as well as high-grade glioma tissue, the TPF signal can be still observed when the MPM imaging depth is over 100 µm (A and C).

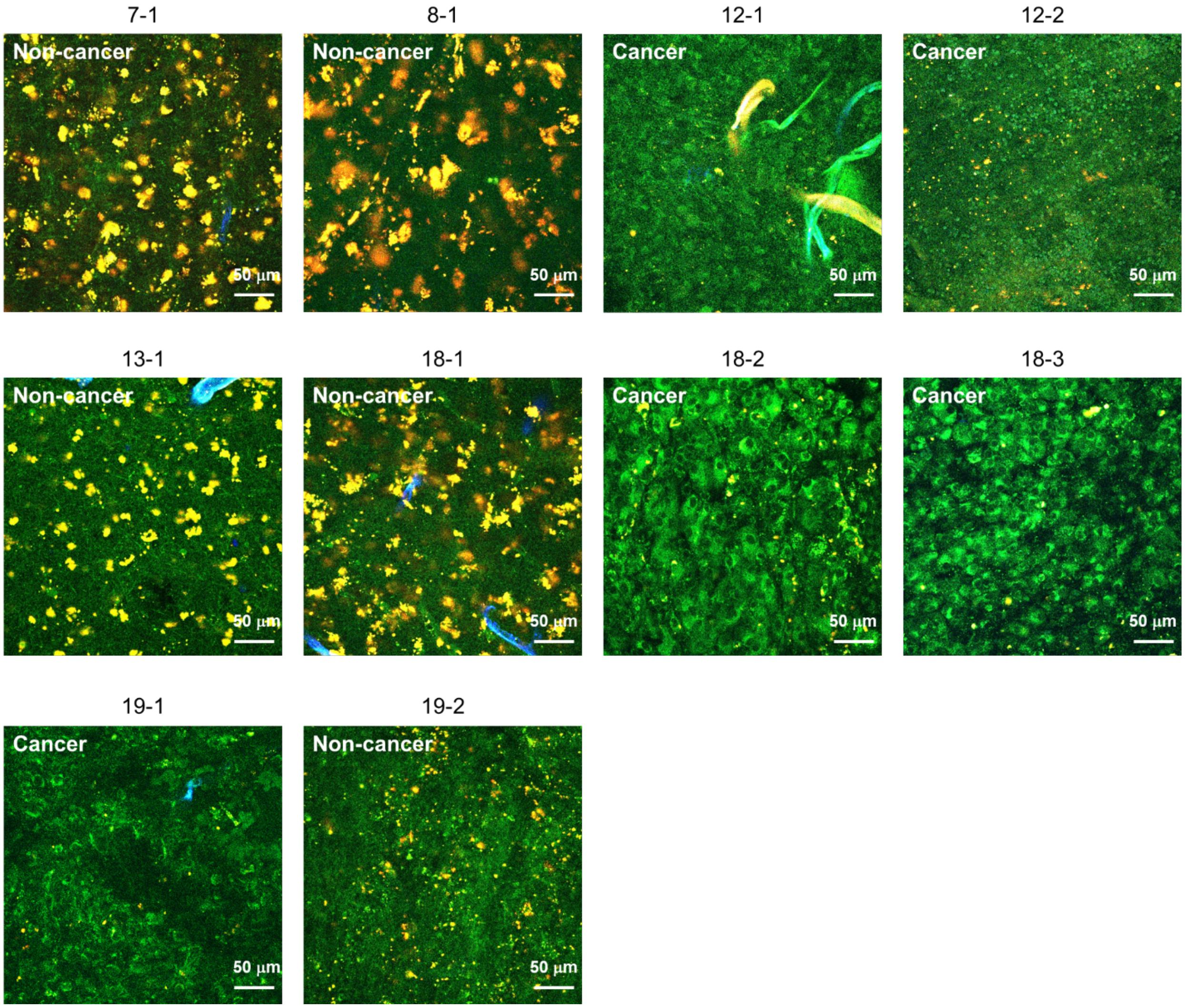

Figure S8 | Representative images of each of the 10 specimens used for classification testing of deep learning of cancer/non-cancer classification network.

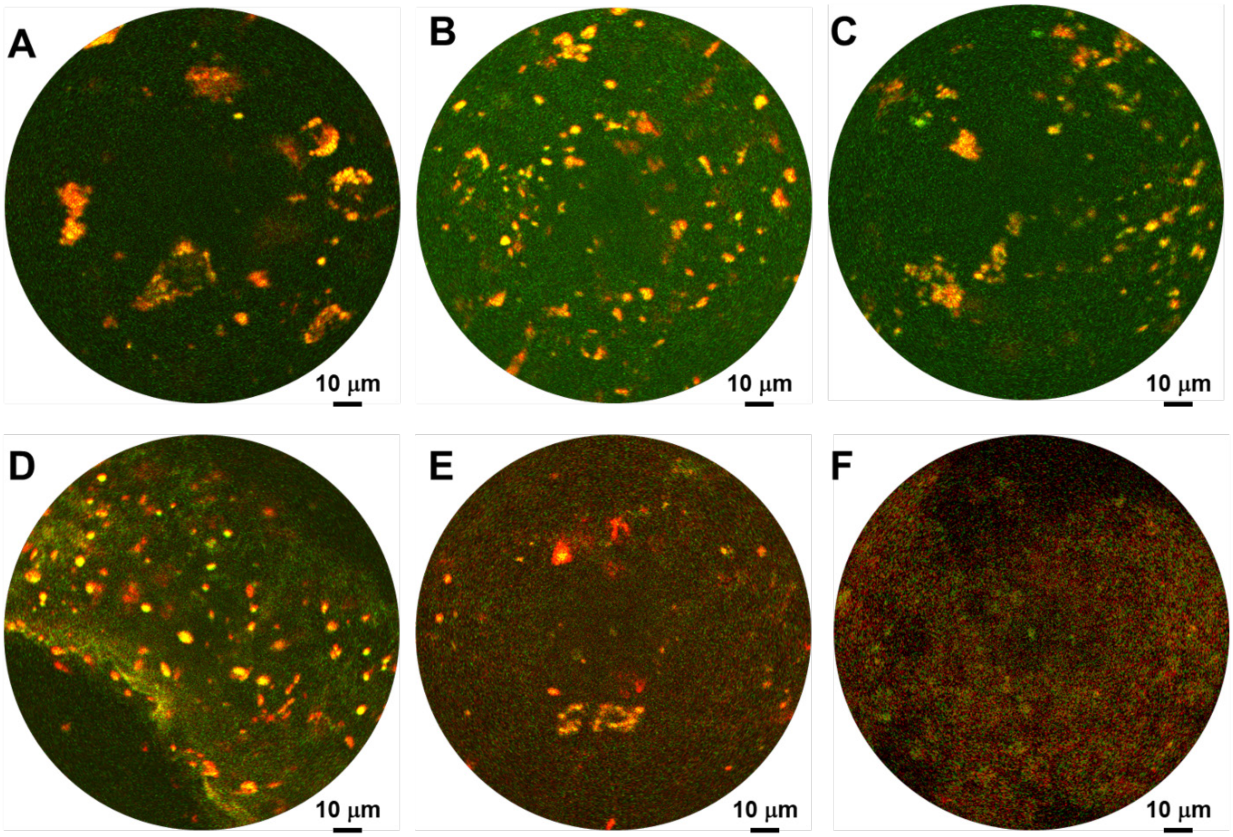

Figure S9 | Single-frame endomicroscopic label-free TPF images of intact human brain tissue. The images shown here are the corresponding single-frame version (frame acquisition time ~0.5 second) of images shown in Figure 6 of the main text, with the same average excitation power (~40 mW at 780 nm). Despite the discernible salt-and-pepper noise, these single-frame images reveal similar structural details seen in the twenty-frame-averaged versions in Figure 6.

Supplementary Tables

Table S1. Clinical and histopathological characteristics of our human samples

#	AGE (YEARS)	SEX	FINAL DIGNAOSIS	TISSUE TYPE	QUANTITY OF SPECIMENS
**1**	40	F	Normal cortex	autopsy	2
**2**	70	M	Normal cortex	autopsy	1
**3**	17	M	Normal cortex	autopsy	1
**4**	80	F	Normal cortex	autopsy	1
**5**	60	F	Normal cortex	autopsy	2
**6**	30	M	Normal cortex	autopsy	2
**7**	70	F	Normal cortex	autopsy	2
**8**	70	F	Normal cortex	autopsy	2
**9**	38	M	High-grade glioma	Fresh-excised	3
**10**	65	M	High-grade glioma	Fresh-excised	4
**11**	50	M	High-grade glioma	Fresh-excised	2
**12**	48	F	High-grade glioma	Fresh-excised	2
**13**	64	M	Metastases	Fresh-excised	1
**14**	84	M	Cerebellar medulloblastoma	Fresh-excised	3
**15**	76	F	High-grade glioma	Fresh-excised	2
**16**	58	M	Recurrent high-grade glioma	Fresh-excised	1
**17**	74	M	High-grade glioma	Fresh-excised	2
**18**	67	M	Recurrent high-grade glioma	Fresh-excised	3
**19**	63	F	High-grade glioma	Fresh-excised	2

Table S2. Summary of key diagnostic histologic features of non-cancerous and cancerous brain tissues

	Features	MPM	H&E histology
**Non-cancer brain tissues**	Neurons	• Dark nuclei<br>• Yellow lipofuscin-like granules in cytoplasm	• Large round nuclei with prominent nucleoli<br>• Cytoplasm slightly different color from neuropil<br>• Yellow lipofuscin granules in cytoplasm
	Glial cells	• Smaller dark nuclei<br>• Fewer lipofuscin-like yellow granules in cytoplasm	• Smaller nuclei
	Axon	• Green linear structures<br>• Interspersed small yellow lipofuscin-like granules	• Pink linear structures
**Brain cancer**	Increased cellularity	• Densely packed cancer cells with dark nuclei	• Densely packed cancer cells often with darkened (hyperchromatic) nuclei
	Nuclear pleomorphism	• Variation in the size and shape of nuclei	• Variation in the size and shape of nuclei
	Microvascular proliferation	• SHG signals from tortuous and disorganized blood vessel wall<br>• Fibrillar collagen deposition in the adventitia of larger blood vessels	• Tortuous blood vessels<br>• Endothelial proliferation
	Collagen deposition	• Strong SHG signals	• Collagen may be less readily identified
	Necrosis	• Characteristic cellular appearance	• Characteristic cellular appearance
